# Pinecone-Inspired Humidity-Responsive Paper Actuators with Bilayer Structure

**DOI:** 10.3390/polym16101402

**Published:** 2024-05-15

**Authors:** David Seelinger, Hussam Georges, Jan-Lukas Schäfer, Jasmin Huong, Rena Tajima, Christan Mittelstedt, Markus Biesalski

**Affiliations:** 1 Ernst-Berl-Institut für Technische und Makromolekulare Chemie, Technical University Darmstadt, Peter-Grünberg-Str. 8, 64287 Darmstadt, Germanyjasmin.huong@hotmail.de (J.H.);; 2 Fachgebiet für Leichtbau und Strukturmechanik, Technical University Darmstadt, Otto-Berndt-Str. 2, 64287 Darmstadt, Germanychristian.mittelstedt@lsm.tu-darmstadt.de (C.M.)

**Keywords:** bio composite, hygroexpansion, cellulose-based actuator, carboxymethyl cellulose

## Abstract

Many plant materials in nature have the ability to change their shape to respond to external stimuli, such as humidity or moisture, to ensure their survival or safe seed release. A well-known example for this phenomenon is the pinecone, which is able to open its scales at low humidity due to the specific bilayer structures of the scale. Inspired by this, we developed a novel humidity-driven actuator based on paper. This was realized by the lamination of untreated paper made from eucalyptus fibers to a paper–carboxymethyl cellulose (CMC) composite. As observed, the hygroexpansion of the composite can be easily controlled by the amount of CMC in the impregnated paper sheet, which, thus, controls the morphologic deformation of the paper bilayer. For a more detailed understanding of these novel paper soft robots, we also studied the dynamic water vapor adsorption, polymer distribution and hygroexpansion of the paper–polymer composites. Finally, we applied a geometrically nonlinear finite element model to predict the bending behavior of paper bilayers and compared the results to experimental data. From this, we conclude that due to the complexity of structure of the paper composite, a universal prediction of the hygromorphic behavior is not a trivial matter.

## 1. Introduction

Plant materials in nature that have the ability to change their form and appearance driven by moisture are called hygromorphs [1]. These naturally occurring plant materials respond to external stimuli, such as humidity or moisture, with the goals of adapting to environmental changes and ensuring their survival [2]. Humidity-driven movement in plants is often based on the differential swelling capacity of structured tissue and its functions, similar to a bimetallic temperature sensor, where temperature is replaced by humidity as the external stimulus. An interesting and well-studied example of this phenomenon is the pinecone with its unique scale pattern [1,3,4]. In the wet state, the cone scales are tightly closed and open incrementally in a low-humidity environment for safe seed release. Since the cone cells are dead, the mechanism of the reversible opening and closing of the cone is a passive behavior. A closer look at the pinecone scales reveals that the plant uses a structural element commonly found in nature for humidity-driven movement: an ordered structure of cellulosic microfibrils in the cell wall within a matrix of hemicellulose and lignin. Here, these microfibrils wind around the central lumen of the cellulose fiber at a specific angle, called the microfibril angle (MFA). This angle influences the swelling direction of the fiber, because microfibrils have a higher swelling degree in the transverse direction compared to the longitudinal direction. In the case of the pinecone, a small region of the scale changes its configuration due to a specific MFA, while the other part of the scale amplifies the movement geometrically. The active region is located at the outer layer of the scale and swells or shrinks, while the inner, passive layer of the scale does not respond to changing humidity in the same manner. This results in the deformation of the tissue due to the mismatch of the hygroscopic expansion between the two layers. In addition, bending stresses and deformations occur in the bilayer structure when it is subjected to a humidity change. The higher the mismatch between the two layers, the greater the deformations in the structure. The geometric shape changes in bilayer systems were first described by Timoshenko [5]. Based on the common laminate theories, the resulting deformation is due to the entries of the laminate stiffness matrix [6,7]. The laminate stiffness matrix, the ABD matrix, consists of three submatrices: the membrane matrix *A*, the plate stiffness matrix *D* and the coupling stiffness matrix *B*. Since the mechanical properties of the bilayer beam are not symmetrical, the entries of the submatrix *B* that signifies bending–extension coupling do not vanish. This coupling may be used to control the structure’s shape under different conditions, as shown by Goo and Yuan et al. [8,9].

Apart from bilayer structures such as the mentioned pinecone, nature also utilizes elastic instability actuation, as, for example, seen in Venus flytraps [10], or the edge growth in petals, like in lily flowers. More data on naturally occurring hygromorphs have recently been reviewed by Poppinga et al. [11]. Since nature already utilizes cellulosic materials for actuators, it is intuitive to consider the use of paper as a starting material for the development of cellulose-based biomimetic composites. Paper consists of a network of cellulose fibers and offers several beneficial characteristics: it has outstanding fluidic properties, and as a porous material, it can take up moisture from the environment, both as vapor and in bulk, the latter via strong capillary forces. With respect to its weight, it has outstanding tensile strength, and still it is bendable and foldable. Finally, it consists of a renewable resource and is recyclable; thus, it is considered a very sustainable material. 

In the recent past, scientists took advantage of the (self-)shaping behaviors of composite materials, for example, by controlling the orientation of reinforcing fibers/particles within the composite material [12,13]. In addition, many well-operating paper-based soft actuators that can change their shapes in response to environmental changes were developed [14,15]. However, these paper-based soft robots have in common the fact that they utilize paper as a simple carrier for stimuli-responsive materials such as bi-metals or smart polymers being attached to the paper [16,17,18].

The hygroexpansion of paper is well known and mainly driven by single-fiber hygroexpansion, which is affected by a variety of factors. Those factors can be divided into single-cellulose-fiber parameters, such as microfibril angle [19,20], fiber length with still controversially discussed observations [21,22] and the thickness of cell wall layers [23], and process parameters such as drying conditions [24], fiber orientation [25,26,27] and the addition of fillers [25], polyelectrolyte or a crosslinked polymer network [28] during the paper-making process. In addition, the source of cellulose fibers is an important factor due to the different amounts and chemical compositions of hemicellulose [29,30], lignin [31,32] and, of course, cellulose itself [32,33,34]. A detailed summary of factors influencing single-fiber and paper-sheet hygroexpansion was recently published by M. Lindner [26,35].

As mentioned before, another option to control the hygroexpansion of paper is the addition of polymers. In the context of hygroexpansion, crosslinkers and crosslinked polymer network are added to paper sheets to ensure dimensional stability for further paper applications such as printing. During the printing process with water-based inks, a through-thickness moisture gradient is unavoidable, which causes fibers to curl out of plane. When this dimensional change in the paper sheet exceeds a critical level, the paper becomes unusable for further printing processes. The addition of crosslinkers and crosslinked polymers to paper sheets is reported to enhance dimensional stability by reducing hygroexpansion due to the increased number of fiber–fiber joints and increased size of the fiber contact area [36]. The reduction in the hygroexpansion of paper has been studied extensively by the scientific community and the paper industry on a macroscopic sheet scale. However, much less is known about the influence of polymers on paper sheets regarding maximizing hygroexpansion. However, the latter may become crucial for the successful development of humidity-responsive actuators based on pure paper-based materials. Moreover, maximizing the bending effect of paper-based actuators requires a comprehensive understanding of the polymer’s impact on hygroexpansion in paper sheets.

To gain a better understanding of maximizing hygroexpansion in paper and develop an all-cellulose-based actuator, our focus in this work is on developing a paper-based actuator where paper itself serves as one of the driving forces for the reversible movement. To achieve this, we employed a functional bilayer setup similar to the pinecone approach by creating a paper bilayer with layers of different hygroexpansion coefficients. This was conducted via the lamination of an untreated eucalyptus paper strip and a paper strip impregnated with CMC. Here, CMC was chosen due to its high water absorption properties and good adhesion to cellulose fibers. Untreated eucalyptus paper and CMC–paper composites should theoretically show different hygroexpansion coefficients in a humid environment and, thus, different expansions and shrinkages, respectively, of the individual paper strips.

## 2. Materials and Methods

### 2.1. Materials

Carboxymethyl cellulose (M_n_ = 124 × 10^3^ g mol^−1^, M_w_ = 333 × 10^3^ g mol^−1^, D = 2.685; degree of substitution 0.7), hydroxypropyl cellulose (Klucel, M_w_ = 1.0 × 10^5^ g mol^−1^ (manufacturer details, no method disclosed), molar substitution (MS) = 5.54 [37]), rhodamine B isothiocyanate and branched polyethylene imine (50% aqueous solution, M_w_ = 750 × 10^5^ g mol^−1^, M_n_ = 60 × 10^5^ g mol^−1^ (manufacturer details, no method disclosed)) were purchased from Sigma-Aldrich (unless otherwise mentioned) and used without further purification.

### 2.2. Formation of Lab-Made Paper Samples

For the formation of lab-made paper samples of eucalyptus fibers, 30 g of air-dried eucalyptus pulp (moisture content 5 wt%) was cut into small pieces of approximately 2 cm^2^ and immersed in 2 L of distilled water over night. The cut eucalyptus pulp was disintegrated with a Estanit AG 04 pulp disintegrator for 75,000 revolutions. This procedure was repeated. The resulting pulp mixtures were combined and diluted with 23 L of water in the pulp distributer during continuous stirring. After this, a series of paper sheets with a grammage of 100 g m^−2^ containing 0.5 wt% of polyamidoamine epichlorohydrin (PAE) as a wet strength agent were made using a Rapid-Koethen hand sheet maker according to DIN 54358 [38]and ISO 5269/2 [39]. The lab-made sheets were stored in a normal climate (23 °C, 50% humidity) for at least 24 h prior to further modification and usage.

### 2.3. Formation of Paper Composite with CMC

**Figure 1:**
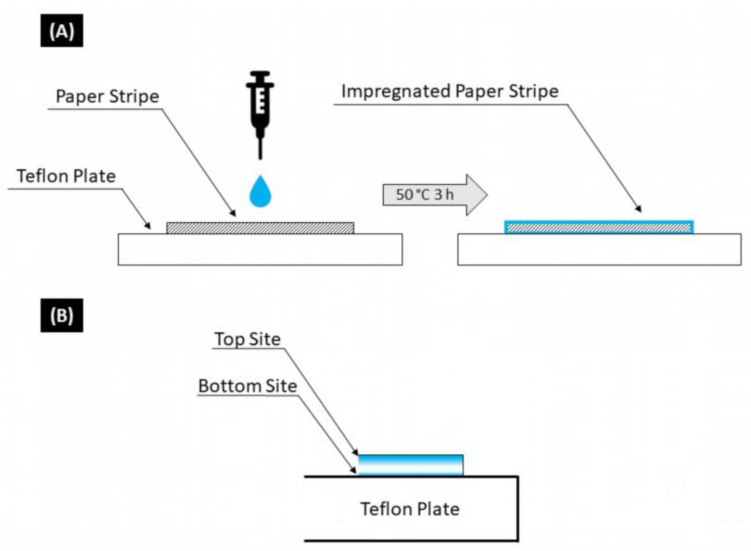
(**A**) Schematic overview of the formation of CMC–paper composites with aqueous CMC solution. (**B**) Schematic overview of polymer gradient formation in the *z*-axis due to polymer transport during the drying of CMC–paper composites.

Before the impregnation of CMC into lab-made paper samples, paper sheets of eucalyptus with a grammage of 100 g m^−2^ (see experimental Section 2.2) were cut into 120 mm × 15 mm samples and stored in a normal climate (23 °C and 50% humidity) for at least 24 h. The average weights of five of these paper samples were determined in a normal climate. Then, 1.5 mL of an aqueous CMC solution (pH = 6) was equally distributed to the paper strips with an Eppendorf^®^ syringe on a Teflon^®^ plate. The amount of CMC impregnated into the paper strips was controlled by the concentration of the aqueous CMC solution to the result in 1.0, 5.0, 10, 20, 30 and 40 wt%, referred to as dry fibers (See Table S1). In the next step, the impregnated paper strips were dried on the Teflon^®^ plate in an oven at 50 °C for 3 h and stored in a normal climate (23 °C and 50% humidity) for at least 24 h until further characterization and usage. Figure 1 shows a schematic overview of the formation of CMC–paper composites. For dynamic vapor adsorption measurements, a multisample system of proUmid with a humidity treatment between 0.5 and 95% RH was used. 

### 2.4. Labeling of CMC with Rhodamine B

CMC was fluorescently labeled according to the literature [40]. In the first step, 1.0 g of CMC (1.0 eq) was dissolved in 100 mL distilled water in a 250 mL round flask with the help of stirring for several hours. In the next step, a solution of 4.2 mg rhodamine–isothiocyanate (RBITC) (M = 536.1 g mol^−1^, 1.6 × 10^−3^ eq) in 210 µL of DMSO was added, and the resulting pink mixture was stirred for 1.5 h at 40 °C. To remove unbound RBITC, the reaction mixture was dialyzed against distilled water with a dialysis tube with a molecular weight cut-off (MWC) of 14 kDa for 6 days. For this, the dialysis tube was immersed in 4.5 L of distilled water and gently stirred. The distilled water was renewed every day, and the progress of the dialyses was monitored via UV-VIS at a wavelength of 575 nm. After 6 days, no unbound RBITC could be detected via UV-VIS, and a slight pink product was collected via freeze drying.

### 2.5. Paper Bilayer Formation

**Figure 2:**
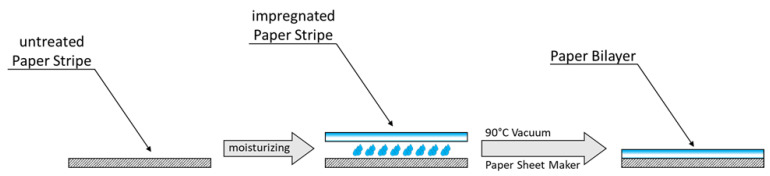
Schematic overview of the formation of paper bilayers made from untreated eucalyptus paper and CMC–paper composites.

Paper bilayers were formed via the impregnation of paper samples made according to experimental Section 2.3. For this, paper strips with and without added CMC were wetted with distilled water with the help of an air brush pistol for approximately 4–5 s. In the next step, the wetted paper strips were stacked, wrapped in baking foil and dried at 90 °C for 10 min with the help of a vacuum using the drying part of the Rapid-Koethen hand sheet maker. During the drying process, the side with the impregnated paper strip faces upwards. The resulting bilayers were stored in a normal climate (23 °C and 50% humidity) for at least 24 h. A schematic overview of the formation of the paper bilayers are shown in Figure 2.

### 2.6. Determination of Hygroexpansion and Young’s Moduli of Paper Samples Impregnated with CMC

For the determination of the hygroexpansion, a series of impregnated paper strips (see Section 2.3) were formed. In the next step, a series of four paper samples per CMC concentration were treated with humidity levels of 10, 50 and 90% RH in a PMMA humidity chamber with an attached humidity controller MGH32 from proUmid for at least 2 h. The humidity treated paper strips were stored for transportation in a closed plastic bag (which was also treated with the respective humidity at the same time), and the paper samples were photographed via a high-resolution scanner. The images were binarized and the area of the surface was calculated via the program ImageJ^®^ version 1.54. For calculation, the images of four paper samples per CMC concentration were used, and the lengths of the paper samples were calculated from the area with the following formula:

$$L=\sqrt{\frac{length\;of\;paper\;sample}{width\;of\;paper\;sample}*Area\;of\;paper\;sample}=\sqrt{8*Area\;of\;paper\;sample}$$

The calculated results of the hygroexpansion coefficients are shown in Figure S1 and were calculated using the determined length changes in the paper samples with the help of the following formula:

$$\alpha =\frac{L_{90\%\;RH}-L_{10\%\;RH}}{80}$$

For the determination of Young’s modulus, the first paper samples with CMC contents of 5 to 40 wt% were prepared as described above in Section 2.3 and stored at 50% RH and 23 °C for at least 24 h. Measurements were determined using a Zwick Roell Z1.0 with a constant strain rate of 10 mm min^−1^, and results are shown in Table S2.

### 2.7. Actuator Measurements—General Procedure

Prior to the actuator measurements, the RH was set with a humidity controller MSG32 (proUmid, Germany) to 50% and the PMMA chamber (Figure S2) was acclimatized without paper bilayers for at least 1 h. Paper bilayers made from two eucalyptus paper strips impregnated with and without CMC (see experimental Section 2.5) were each attached to a clamp and placed in the PMMA Box. In the next step, the RH was set to 50% for 30 min, followed by 70%, 90%, 70%, 50% 30% and 10% for 2 h each. After reaching 10% RH, the humidity was increased again to 30%, 50% 70% and 90% RH, also for 2 h each (for the full RH program, see Figure S3). This procedure was repeated once (excluding the prior 50% RH for 30 min at the start). The displacement of the paper bilayers was captured every 10 min with a vertical attached Epson D600 camera.

## 3. Results

### 3.1. Formation of CMC–Paper Composites

For the formation of CMC–paper composites, eucalyptus paper is impregnated with an aqueous CMC solution and dried afterwards on a Teflon^®^ plate in an oven, as described in Section 2.3. During the drying of the CMC–paper composite, i.e., the evaporation of the solvent, polymer mass transport takes place [41]. Since paper strips impregnated with CMC are placed on a Teflon plate during the drying process, most of the water evaporation takes place only on the upper side of the paper strip. Capillary forces then continue to transport water and, thus, CMC to the upper side of the paper strip. This results in a polymer gradient along the *z*-axis, as shown in principle in Figure 1B.

Therefore, we investigate this polymer gradient in the paper layers first by conducting image analysis of the cross-section of CMC–paper composites with the help of fluorescent confocal laser scanning microscopy (CLSM). Untreated eucalyptus paper samples are stained with calcofluor white (CFW) prior to CMC impregnation. This dye is known to bind specifically to cellulose surfaces, allowing distinction between cellulose fiber network and CMC. In the next step, these paper strips are impregnated with rhodamine B isocyanate (RBITC) labeled CMC in the same way as described in Section 2.4 and embedded in polyurethane resin (PU) to obtain paper slices after cutting with a thickness of 54 µm.

**Figure 3:**
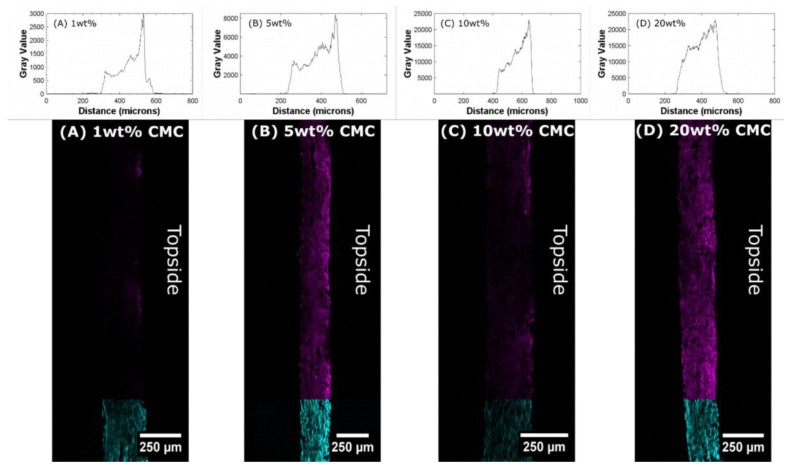
Cross-section of paper samples impregnated with RBITC-CMC and subjected to prior impregnation with CFW for better distinction of cellulose fiber network (cyan) and incorporated CMC (purple) concentration of 1 to 20 wt% (**A**–**D**). The right side of the cross-section shows the top side during the drying process. Above the CLSM images, the corresponding gray value plots show the CMC distribution in the cross section.

These thin paper samples enable the investigation of the polymer gradient in cross-section of the paper via fluorescent CLSM and are shown in Figure 3A–C. These CLSM images and the grey values obtained from the images for paper samples have respective CMC contents of 1 wt%, 5 wt%, 10 wt% and 20 wt% relative to the weight of the sample. The related grey values plotted above the CLSM images are showing the CMC distribution in the cross section.

It can be inferred from the images and the respective grey value plots that a CMC z-gradients are formed in the paper strips. As mentioned before, the gradient originates from transport effects due to capillary forces during drying on a Teflon^®^ plate. If CMC concentrations of less than 20 wt% relative to the fiber weight are applied, the labeled CMC is located on the upper side of the paper sample. In contrast, when the CMC concentration is increased to 20 wt%, the formation of a polymer gradient becomes less pronounced (see Figure 3D). This is attributed to the high viscosity of the CMC solution, which slows down the otherwise very fast transport of CMC to the upper side of the paper. The removal of water during drying enhances this effect even further. Due to the formed polymer gradient in the paper samples, a difference in hygroexpansion and, therefore, bending behavior in an environment with changing humidity is expected.

### 3.2. Dynamic Vapor Sorption of CMC–Paper Composites

**Figure 4:**
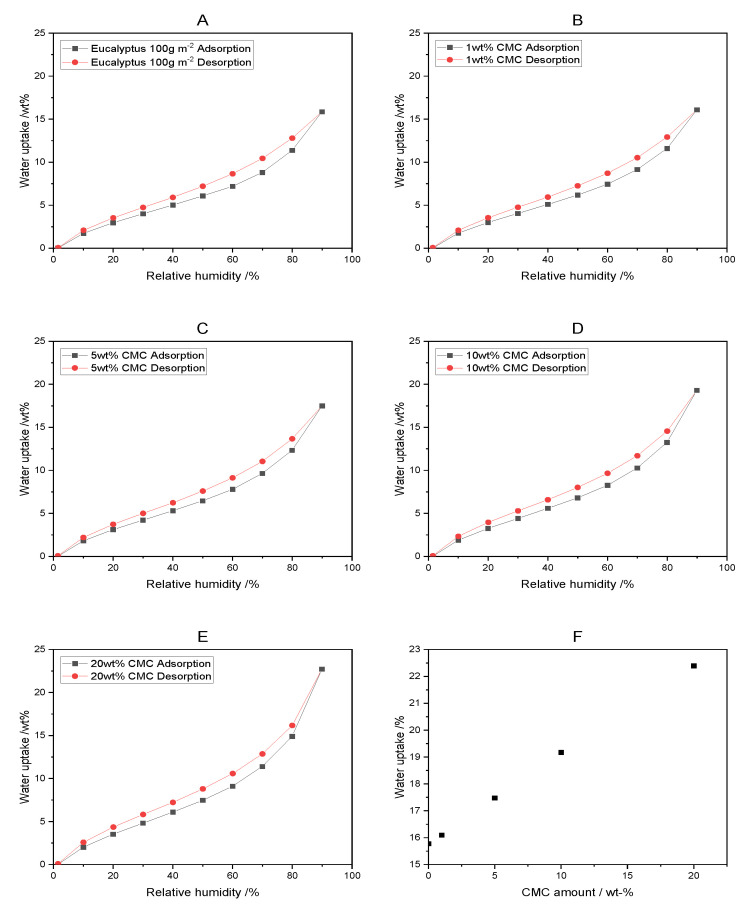
Dynamic water vapor adsorption and desorption, respectively, of eucalyptus paper samples with varying CMC contents (**A**–**E**) and water uptake levels at 90% RH for paper–CMC composites. Graph (**F**) shows the water uptake at 90% RH for CMC–paper composites.

Next, the water vapor sorption characteristics of the functionalized paper are considered. We study the water vapor adsorption of our lab-made eucalyptus paper samples impregnated with CMC at different humidities via dynamic vapor sorption (DVS). A rising amount of CMC in eucalyptus paper samples results in increased water adsorption at high humidity, as is shown in Figure 4A–E. Here, water adsorption increases linearly to 22.40 wt% (Figure 4F) for eucalyptus paper impregnated with 20 wt% CMC at 90% RH compared to a 15.78 wt% increase for untreated eucalyptus paper, in agreement with the literature [42]. 

In addition, CMC–paper composites show hysteresis during the adsorption and desorption of water (Figure 4), which is common for paper and reported in the literature [43]. The increase in water adsorption can be explained by the high water absorption capacity of CMC (57.9 wt% increase at 90% RH, see Figure S4). Hence, we hypothesize that eucalyptus paper samples impregnated with CMC have a higher hygroexpansion compared to untreated paper. For cellulosic materials, water vapor uptake correlates with hygroexpansion, as has been shown by others [44].

### 3.3. Hygroexpansion of Paper Samples Impregnated with CMC

To confirm this hypothesis of hygroexpansion, we conducted experiments to measure the hygroexpansion of eucalyptus paper strips impregnated with carboxymethyl cellulose (CMC) using image analysis. The hygroexpansion of the paper samples impregnated with CMC was determined after a humidity treatment consisting of the following steps: 50%–90%–50%–10%–50%–90%–50%–10%–50% for at least 2 h each. After each humidity step, the paper samples were analyzed using a high-resolution scanner. The resulting images of four paper samples per CMC concentration were converted into binary images, and the area was calculated using the ImageJ^®^ program. For the calculation of the hygroexpansion, the images of four paper samples per CMC concentration were used, and the length of the paper samples was calculated from the area with the formula described in Section 2.6. It is important to note that the respective lengths of the hygroexpansion measurements here only detect the length change due to the 2D capturing of the images. Changes in paper thickness are, therefore, not measured. 

**Figure 5:**
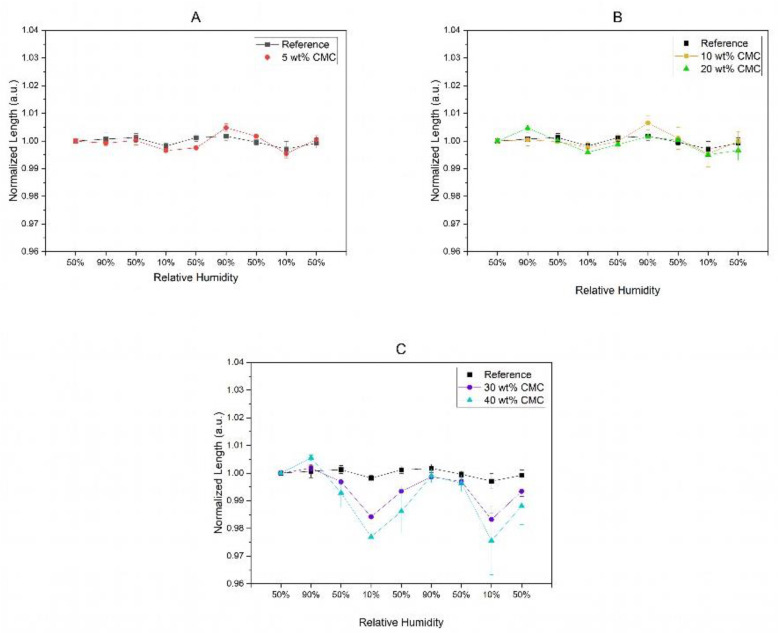
Hygroexpansion of paper samples impregnated with CMC concentration of 5 wt% (**A**), 10 wt% and 20 wt% (**B**) and 30 wt% and 40 wt% (**C**), respectively. Untreated paper strips were used as a reference.

Figure 5 shows the normalized length (dotted lines) of the paper samples as a function of the relative humidities, respectively, after various humidity cycles between 90% and 10% RH. The applied RH values are superimposed on the data as blue columns. If paper composites with 5 wt% and 10 wt% CMC are investigated, no significant change in length can be observed. However, the lengths of paper samples with higher concentrations of CMC of 20 wt% to 40 wt% are behaving differently. Here, the lengths of paper strips with high amounts of impregnated CMC are slightly increasing by a relative change of less than 1% compared to the untreated reference when the relative humidity is increased from 50% RH to 90% RH. The latter is due to absorbing water vapor and the subsequent swelling of the CMC–cellulose fiber composite material. Still, this length change of approx. 0.6% (for 40 wt% CMC) is considerably larger compared to the relative length change in the reference samples, which is about 0.07%. These observed results are in good agreement with those values reported by others [28,45]. With the help of the determined length changes, we calculated the hygroexpansion coefficients of paper samples impregnated with varying CMC concentrations, as shown in Figure S1 and described in Section 2.6. Furthermore, with the help of the determined hygroexpansion coefficients, it is possible to calculate the deflection of paper bilayers in a humid environment. This calculation is based on the common laminate theory, as mentioned already, and is summarized at the end of this work in Section 3.4.

As Figure 5 shows, the effect of the length change becomes much more pronounced when the RH is decreased to 10%. Paper samples with high concentrations of CMC showed length changes of about 2.5% (in the case of 40 wt% CMC) and about 1.5% (in the case of 30 wt% CMC) at 10% RH compared to the significantly less pronounced hygroexpansion of native paper samples. Thus, a reduction in humidity has a significantly stronger impact on the changes in the lengths of the paper strips compared to the untreated paper samples. CMC molecules located in the macro pores of the paper network and pre-swollen in high humidity can increase fiber–fiber bond strength by increasing the relative bonded area similar to other macromolecules [46,47]. If CMC is shrinking due to a humidity decrease, paper fibers connected to the swollen CMC-chains in the macropores are pulled together, resulting in an overall reduction in length. 

**Figure 6:**
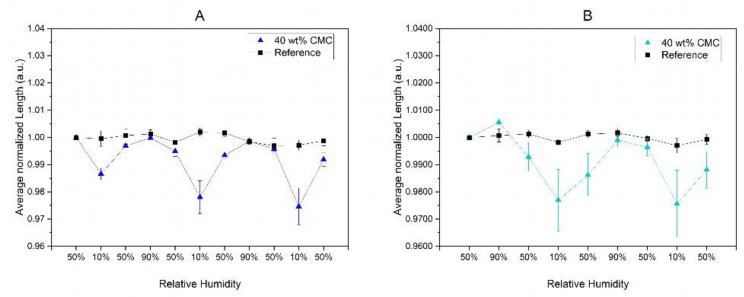
Length changes due to the hygroexpansion of paper strips impregnated with 40 wt% CMC. When paper strips after acclimatization at 50% RH were first exposed to high humidity of 90% (**A**), the change in length at low humidity of 10% is significantly larger compared to paper strips treated with 10% RH without prior treatment at 90% RH (**B**).

We further observe that paper strips with concentrations of 40 wt% CMC that were first exposed to 10% RH show a relative change in length of ~1.3%, while the same samples treated with a humidity of 90% beforehand show an increasing relative change in length of ~2.7% (Figure 6). This observed behavior of the paper strips can be explained by the initial filling of the macropores with CMC and the subsequent contraction of the polymer network at low humidity, as hypothesized above. Therefore, the initial treatment of paper strips with high humidity of 90% RH is crucial for maximizing the possible hygromorphic humidity responses of the paper strips.

### 3.4. Humidity-Responsive Movement of Paper Bilayers

Next, we are interested in studying laminated paper bilayer composites and their respective hygromorphic behaviors. To do so, we laminate untreated paper to a CMC–paper composite. This results in a paper bilayer with layers of different hygroexpansion properties. For lamination, the layers are moisturized via water mist and afterwards dried on top of each other under paper-making condition, as shown in Figure 2. To ensure the stability of the paper sheets in the wet state during lamination, all lab-made paper sheets are strengthened with 0.5 wt% polyamidoamine epichlorohydrin as a wet strength agent during the paper-making process, as described in the experimental Section 2.2. Since the previously conducted hygroexpansion measurements conclude a significant shrinking of the paper length of CMC–paper composites compared to untreated paper, this combination of paper layer should result in a bending behavior when the humidity is decreased.

**Figure 7:**
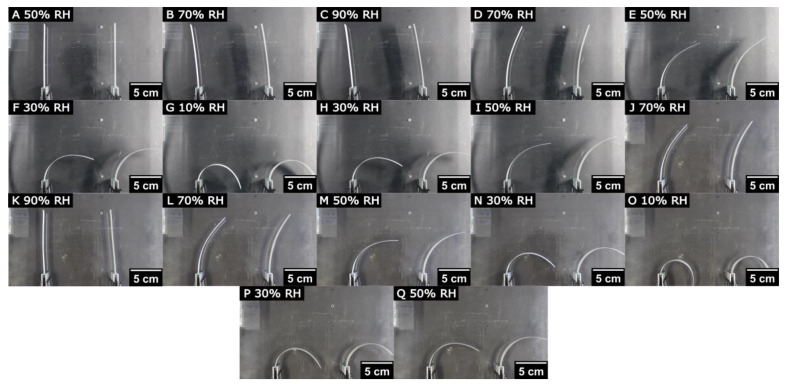
Humidity-responsive bending of paper bilayers with a CMC–paper layer with 40 wt% CMC. Here, paper bilayers are shown from a top view. For reproducibility, two samples are investigated at the same time. The first images (**A**–**C**) show the initial wetting phase with high humidity. In the next step (**D**–**G**), the humidity is decreased subsequently to 10% RH, resulting in the bending of the paper bilayers. In the following images (**H**–**P**), the sequence of humidity treatment is repeated, stopping at the initial settings of 50% RH (**Q**).

To investigate the bending behavior of paper-based bilayers in response to humidity, two identical paper bilayers are placed in a polymethylmethacrylate (PMMA) box equipped with a humidity controller with the help of clamps, as shown in Figure S2, and the environmental conditions inside the PMMA box are adjusted with an attached humidity controller. The humidity treatment involves a sequence of humidity levels after starting at 50% RH, then rising stepwise to 90% RH, followed by a stepwise decrease to 10%. This humidity treatment is repeated once (the detailed RH program is described in Section 2.7 and shown in Figure S3). Due to the different hygroexpansion coefficients, the paper bilayers are bending during the humidity treatment, as shown in Figure 7. Here, the side view of the paper layers is shown with the CMC–paper layer positioned on the right side and the untreated paper layer on the left side. Based on the hygroexpansion experiments that indicate significant benefits for the bending movement when the paper layers are subjected to high humidity (90% RH) prior to dehydration at 10% RH, the humidity treatment starts with an increase in humidity to 90%.

**Figure 8:**
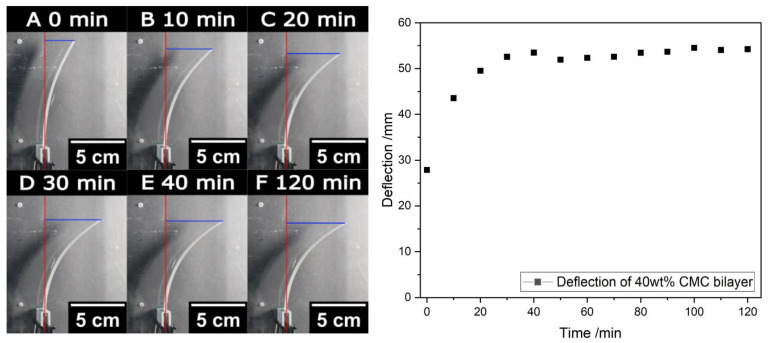
Humidity-responsive movement during the change from 70% to 50% RH of paper bilayer with a CMC–paper layer with 40 wt% CMC after different times (**A**–**F**). The deflections of the paper actuators were calculated using the red and blue lines. The major part of bending happens in the first 20 min.

Due to the water uptake of the CMC and, thus, the expanding length of the CMC–paper layer, the bilayers first bend slightly to the left side when treated with high humidity. As mentioned before, we hypothesize that the swollen CMC macromolecules fill the macropores of the paper network first and, therefore, only slightly change the layer length and bending of the paper bilayer. After treatment at 10% RH, the humidity inside the chamber is returned to 90% RH successively, causing the paper strips to return to their original positions. Note that the original position of the paper bilayer is not reached at a humidity of 50% but only at a rewetting at 90% RH. The images show the paper bilayers in the equilibrium state at the end of each humidity step. To ensure the equilibrium state of bilayer bending, the time for every humidity step was set to 2 h. However, the actual bending movement only takes several minutes, as shown in Figure 8. Here, the deflection of the paper strip is determined via image analysis and shows that the bending movement of the paper bilayers during the humidity change of 70% to 50% RH is already finished after approximately 30 min, highlighting the rapid response of this bilayer material. To further improve the response times of paper bilayers, future studies will focus on the time dependence of the bending behavior.

**Figure 9:**
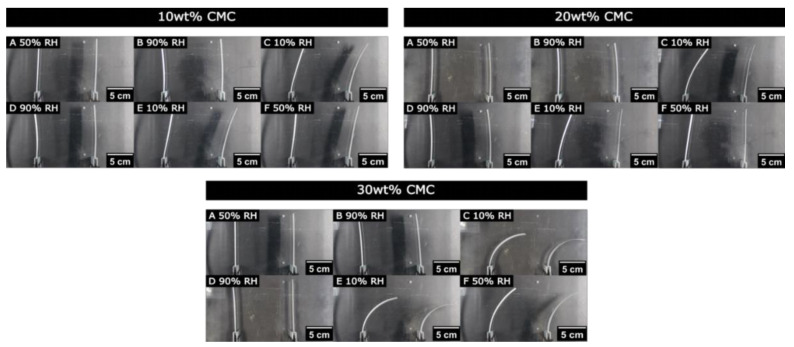
Bending behavior of paper bilayers with varying CMC concentrations given in inserts above the photographs. The relative humidities denoted as inserts to individual photographs were varied from 50% (A, F) to 90% (B, D) and 10% (C, E), respectively.

In the next step, we control the bending behavior of the paper bilayer by incorporating varying amounts of CMC in the bilayers. This is achieved by adjusting the polymer concentration of the coating solution utilized during the formation of the CMC–paper layers. As shown in Figure 9, the bending and deflection of the paper bilayers varies with different CMC contents due to the dependence of the hygroexpansion coefficient on increasing CMC content. Paper bilayers are all treated with the same humidity program, as described before (see Section 2.7 and Figure S3, respectively). For a better overview, only the highest deflections of the bilayers at 90% and 10% RH are shown. Consistent with the hygroexpansion experiments in Section 3.3, the highest deflections are observed at 30 and 40 wt% CMC (shown in Figure 7) contents at low humidity of 10%. Note that all bilayers are able to reach their original position after the treatment with high humidity of 90%. In addition, we investigate two samples at the same time to show reproducibility.

**Figure 10:**
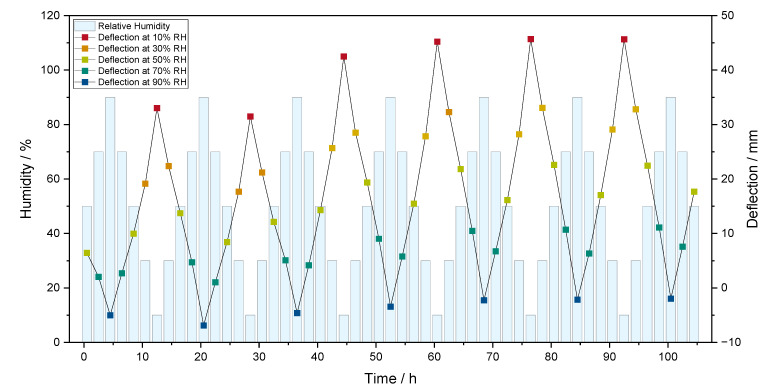
Displacement of the paper bilayers made paper bilayers were made with a 10 wt% CMC paper strip after several humidity cycles between 10% and 90% RH.

To investigate the reversibility of the bilayers after several humidity cycles, the humidity is changed between 50 and 90% RH (A–F) repeatedly. Here, the investigated paper bilayers were made with a 10 wt% CMC paper strip. For this purpose, the deflection to the starting position of the paper bilayer at the respective humidity levels is determined via image analysis, as shown in Figure S5. As shown in Figure 10, the paper bilayer is bending reversibly even after several humidity cycles. However, the initial position at 90% RH of the paper bilayer is not fully achieved and deviates approximately 5 to 6 mm.

At a closer look, the deflection in dry-environment-responsive humidity increases after the first three humidity cycles and then remains constant. The reason for this is the previously applied preload and drying stress during the drying process of the bilayer formation, which is resolved after the first wetting process at high humidity. The position of the paper bilayer at high humidity (90% RH) is influenced in the same manner. Note that the bilayers show no deflection at the start of the humidity treatment at 50% RH, while in the following humidity cycles, the bilayers are bent at 50% RH.

For visionary application of these novel humidity-responsive materials, such as smart wound dressing in medical technology or adaptive shades in the construction industry, it is important to reinforce the paper bilayer materials to withstand environmental influences such as bacterial activity, decay and moisture. The latter is particularly critical due to the susceptibility of paper strength to moisture. Only approximately 5% of the original dry strength upon wetting remain, causing the increased flexibility of the paper fibers [48]. Consequently, the humidity responsiveness of paper bilayers is compromised, as the forces responsible for the bending behavior are unable to be effectively directed. Furthermore, the fiber network could ultimately fail over an extended time period and tensile stress in combination with high moisture.

**Figure 11:**
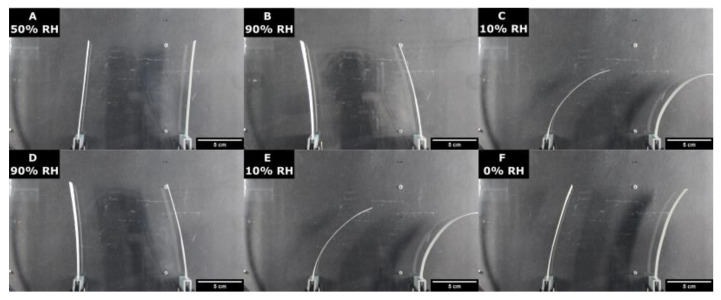
Humidity-responsive bending of paper bilayers made with a 40 wt% crosslinked keto–HPC-–paper strip. The relative humidities denoted as inserts to individual photographs were varied from 50% (A, F) to 90% (B, D), and 10% (C, E), respectively.

To overcome this problem, we next substitute CMC for a more recently developed system based on oxidized hydroxypropyl cellulose (keto–HPC) crosslinked with polyamines. This polymer network exhibits great improvement in (wet) tensile strength once applied to paper, while it is still considered to be hydrophilic (i.e., hydrogel properties). Keto–HPC is made via the TEMPO-oxidation of HPC and is able to crosslink with a variety of amines to form a water-stable network. Here, keto–HPC and polyethylene imine are applied to eucalyptus paper strips, as described in Section 2.2, but without the usage of PAE and a carbonyl to amine ratio of 1.0, as described in our earlier work [37]. Subsequently, paper bilayers are formed, as described previously. As shown in Figure 11, paper bilayers impregnated with 40 wt% crosslinked keto–HPC shows similar bending behavior to CMC, while it additionally wet-strengthens the paper fiber network, as shown before [37].

**Figure 12:**
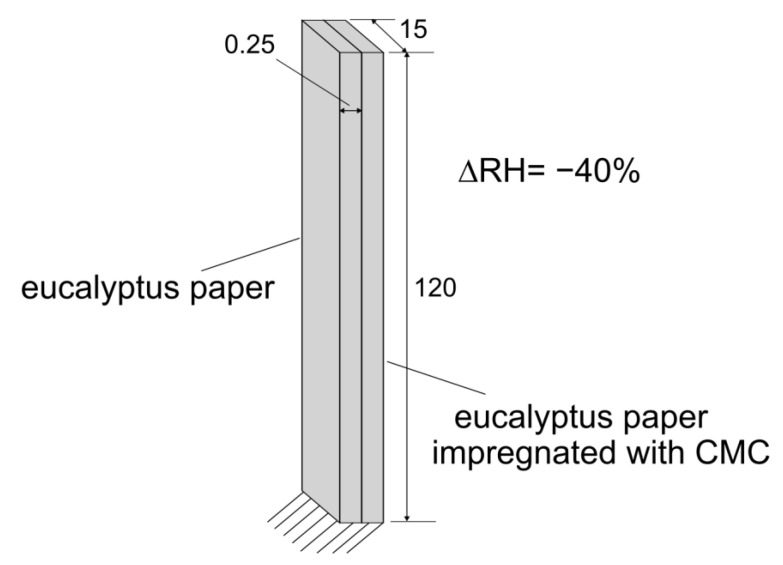
Illustration of the FE model with the dimensions of the paper strip in mm.

As the last investigation, we conduct quasi-static finite element analysis (FEA) for the CMC–paper bilayers with a varying CMC contents of 5 to 40 wt% using Young’s moduli summarized in Table S2. The hygroscopic expansion coefficients for the eucalyptus paper and the paper impregnated by different CMC concentrations are determined using the displacements measured in experiments conducted in this work and are shown in Figure S1. FEA analysis is performed for a cantilever model using the FEA software ABAQUS 2022. The finite element model (FE model) consists of two uniform-thickness strips with the dimensions (length: 115 mm, width: 15 mm, thickness: 0.25 mm) illustrated in Figure 12.

**Figure 13:**
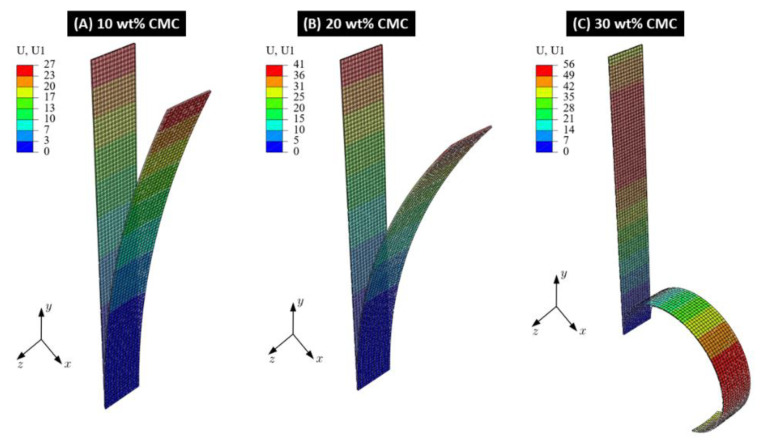
Deflection of paper bilayers impregnated with 10, 20 and 30 wt% CMC at 10% RH.

The strip length in the FE model was reduced by 5 mm to match the experimental set up. One strip is made of the eucalyptus paper without CMC. The eucalyptus paper impregnated with CMC is used for the second strip. Different configurations are considered, where the concentration of the CMC layer varies from 5 wt% to 30 wt%. The materials are assumed to be linear isotropic and exhibit the Poisson ratio *ν* = 0. The strips in the FE model were subjected to a moisture change of ∆RH = −40%. During the simulation for each CMC concentration, the hygroscopic expansion coefficients were assumed to be constant. Three-dimensional quadratic continuum elements (C3D20R) are used to mesh the strips in the 3D finite element model. The mesh size is set to 2 mm so that the full mesh involves 928 elements. As in the experiments, the bottom side of the FE model is clamped. The boundary conditions and the dimensions of the FE model are illustrated in Figure 13. Since large deformations are observed during the experiments, geometric nonlinear behavior is considered in the FEA. Furthermore, self-contact is considered in the finite element model. 

For comparison of experimental and FEA results, Figure 13 shows the deflection determined via the FE model of paper bilayers impregnated with 10, 20 and 30 wt% CMC, respectively. Here, the bending behavior of the paper bilayer with 10 wt% CMC can be described reasonably using the FE model. Here, the deviation from the observed experimental results to the FEA results is approx. 15%. However, this deviation increases with CMC concentrations above 20 wt% due to the nonlinear increase in the determined hygroexpansion coefficient (Figure S1). Therefore, the FE analysis for strips with CMC concentrations above 20 wt% reveals that the paper strips start curling up to form a circle. In contrast, only a semicircle shape is observed in the experiment. The rolling behavior cannot be obtained from simulations with shell elements. While the numerical predictions and the experimental data show identical trends for the CMC concentration 10 wt%, the assumption of the simulation of a moisture-independent hygroscopic expansion coefficient is no longer sufficient at CMC concentrations of 20 wt% and above. An accurate numerical simulation of the paper bilayers considered here requires a deeper investigation into the moisture-independent material properties, which is outside the scope of this investigation and planned for the future. 

## 4. Conclusions

In conclusion, we developed a novel humidity-driven actuator made from paper–polymer composites. In order to accomplish this, untreated eucalyptus paper strips were laminated to eucalyptus paper strips impregnated with CMC or crosslinked keto-HPC. The resulting paper bilayers were able to bend in humid environment due to the different hygroexpansion coefficients of the paper bilayers, similar to the thermal expansion of bimetals. By changing the incorporated CMC mass in the paper layer, the hygroexpansion and the bending behavior in a humid environment is controllable. Here, low humidity levels have a significantly stronger impact on the change in the lengths of paper strips and the bending of the paper bilayer compared to treatment with high humidity. Nevertheless, the initial treatment of paper strips with high humidity of 90% RH is crucial for maximizing the humidity-responsive movement of the paper strips. Due to the excellent reversible bending behavior of CMC–paper bilayers, these paper bilayers could be interesting, for example, in visionary applications such as adaptive shades in facades construction. Further studies will focus on dynamics of the bending in order to obtain a more comprehensive picture of the hygromorphic properties of paper bilayers.

## Data Availability

Data are contained within the article and supplementary materials.

## Supplementary Materials

The following supporting information can be downloaded via this link: https://www.mdpi.com/article/10.3390/polym16101402/s1, Figure S1: Hygroexpansion coefficients of eucalyptus paper impregnated with varying amounts of CMC; Figure S2: Setup of the PMMA humidity chamber. Paper bilayers are attached to staple clips and arranged horizontally to the bottom of the humidity chamber; Figure S3: Set and read humidity levels during the humidity treatment for the investigation of the bending behavior of paper bilayers; Figure S4: Determination of the dynamic water vapor adsorption and desorption of CMC; Figure S5: Bending behavior of a paper bilayer made from untreated eucalyptus paper strip laminated to a paper strip with a polymer content of 10 wt% CMC after several humidity cycles; Table S1: Summary of applied concentrations of aqueous CMC solution to paper samples for the formation of paper–CMC composites; Table S2: Young’s moduli of paper bilayers with varying CMC polymer contents.

## References

[1] Reyssat E., Mahadevan L. (2009). Hygromorphs: From Pine Cones to Biomimetic Bilayers. J. R. Soc. Interface.

[2] Fratzl P., Barth F.G. (2009). Biomaterial Systems for Mechanosensing and Actuation. Nature.

[3] Dawson C., Vincent J.F.V., Rocca A.-M. (1997). How Pine Cones Open. Nature.

[4] Eger C.J., Horstmann M., Poppinga S., Sachse R., Thierer R., Nestle N., Bruchmann B., Speck T., Bischoff M., Rühe J. (2022). The Structural and Mechanical Basis for Passive-Hydraulic Pine Cone Actuation. Adv. Sci..

[5] Timoshenko S. (1925). Analysis of Bi-Metal Thermostats. J. Opt. Soc. Am..

[6] Mittelstedt C. (2023). Theory of Pates and Sells.

[7] Mittelstedt C., Becker W. (2016). Strukturmechanik ebener Laminate.

[8] Goo B., Hong C.-H., Park K. (2020). 4D Printing Using Anisotropic Thermal Deformation of 3D-Printed Thermoplastic Parts. Mater. Des..

[9] Yuan C., Ding Z., Wang T.J., Dunn M.L., Qi H.J. (2017). Shape Forming by Thermal Expansion Mismatch and Shape Memory Locking in Polymer/Elastomer Laminates. Smart Mater. Struct..

[10] Forterre Y., Skotheim J.M., Dumais J., Mahadevan L. (2005). How the Venus Flytrap Snaps. Nature.

[11] Poppinga S., Correa D., Bruchmann B., Menges A., Speck T. (2020). Plant Movements as Concept Generators for the Development of Biomimetic Compliant Mechanisms. Integr. Comp. Biol..

[12] Amjadi M., Sitti M. (2018). Self-Sensing Paper Actuators Based on Graphite-Carbon Nanotube Hybrid Films. Adv. Sci..

[13] Li Q., Le Duigou A., Kumar Thakur V., Liu L., Leng J., Scarpa F. (2023). Three-Dimensional Water Diffusion and Modelling of Flax/Shape Memory Epoxy Composites. Compos. Part Appl. Sci. Manuf..

[14] Liu Y., Shang S., Mo S., Wang P., Yin B., Wei J. (2021). Soft Actuators Built from Cellulose Paper: A Review on Actuation, Material, Fabrication, and Applications. J. Sci. Adv. Mater. Devices.

[15] Ryu J., Tahernia M., Mohammadifar M., Gao Y., Choi S. (2020). Moisture-Responsive Paper Robotics. J. Microelectromechanical Syst..

[16] Weng M., Tang Z., Zhu J. (2021). Multi-Responsive Soft Paper-Based Actuators with Programmable Shape-Deformations. Sens. Actuators Phys..

[17] Hu Y., Xu A., Liu J., Yang L., Chang L., Huang M., Gu W., Wu G., Lu P., Chen W. (2019). Multifunctional Soft Actuators Based on Anisotropic Paper/Polymer Bilayer Toward Bioinspired Applications. Adv. Mater. Technol..

[18] Hu Y., Qi K., Chang L., Liu J., Yang L., Huang M., Wu G., Lu P., Chen W., Wu Y. (2019). A Bioinspired Multi-Functional Wearable Sensor with an Integrated Light-Induced Actuator Based on an Asymmetric Graphene Composite Film. J. Mater. Chem. C.

[19] Burgert I., Frühmann K., Keckes J., Fratzl P., Stanzl-Tschegg S. (2004). Structure-Function Relationships of Four Compression Wood Types: Micromechanical Properties at the Tissue and Fibre Level. Trees.

[20] Gindl W., Gupta H.S., Schöberl T., Lichtenegger H.C., Fratzl P. (2004). Mechanical Properties of Spruce Wood Cell Walls by Nanoindentation. Appl. Phys. A.

[21] Antonsson S., Mäkelä P., Fellers C., Lindström M.E. (2009). Comparison of the Physical Properties of Hardwood and Softwood Pulps. Nord. Pulp Pap. Res. J..

[22] Uesaka T., Moss C. (1997). Effects of Fibre Morphology on Hygroexpansivity of Paper*—*A Micromechanics Approach. The Fundametals of Papermaking Materials.

[23] Pulkkinen I., Fiskari J., Alopaeus V. (2009). The Effect of Hardwood Fiber Morphology on the Hygroexpansivity of Paper. BioResources.

[24] Nielsen I., Priest D. (1997). Dimensional Stability of Paper in Relation to Lining and Drying Procedures. Pap. Conserv..

[25] Figueiredo A.B., Magina S., Evtuguin D.V., Cardoso E.F., Ferra J.M., Cruz P. (2016). Factors Affecting the Dimensional Stability of Decorative Papers under Moistening. BioResources.

[26] Kulachenko A., Gustafsson P.-J., Coffin D.W., Hägglund R., Mäkelä P., Nygards M., Östlund S., Uesaka T., Niskanen K., Berglund L. (2011). Mechanics of Paper Products.

[27] Lavrykov S.A., Ramarao B.V., Lyne O.L. (2004). The Planar Transient Hygroexpansion of Copy Paper: Experiments and Analysis. Nord. Pulp Pap. Res. J..

[28] Larsson P.A., Wågberg L. (2008). Influence of Fibre–Fibre Joint Properties on the Dimensional Stability of Paper. Cellulose.

[29] Belbekhouche S., Bras J., Siqueira G., Chappey C., Lebrun L., Khelifi B., Marais S., Dufresne A. (2011). Water Sorption Behavior and Gas Barrier Properties of Cellulose Whiskers and Microfibrils Films. Carbohydr. Polym..

[30] Marklund E., Varna J. (2009). Modeling the Hygroexpansion of Aligned Wood Fiber Composites. Compos. Sci. Technol..

[31] Reina J.J., Domínguez E., Heredia A. (2001). Water Sorption-Desorption in Conifer Cuticles: The Role of Lignin. Physiol. Plant..

[32] Yamamoto F., Sassus F., Ninomiya M., Gril J. (2001). A Model of Anisotropic Swelling and Shrinking Process of Wood. Wood Sci. Technol..

[33] Joffre T., Neagu R.C., Bardage S.L., Gamstedt E.K. (2014). Modelling of the Hygroelastic Behaviour of Normal and Compression Wood Tracheids. J. Struct. Biol..

[34] Wang N., Liu W., Lai J. (2014). An Attempt to Model the Influence of Gradual Transition between Cell Wall Layers on Cell Wall Hygroelastic Properties. J. Mater. Sci..

[35] Lindner M. (2018). Factors Affecting the Hygroexpansion of Paper. J. Mater. Sci..

[36] Eriksson M., Torgnysdotter A., Wågberg L. (2006). Surface Modification of Wood Fibers Using the Polyelectrolyte Multilayer Technique: Effects on Fiber Joint and Paper Strength Properties. Ind. Eng. Chem. Res..

[37] Seelinger D., Trosien S., Nau M., Biesalski M. (2021). Tailored Oxidation of Hydroxypropyl Cellulose under Mild Conditions for the Generation of Wet Strength Agents for Paper. Carbohydr. Polym..

[38] DIN 54358-1:1981-02 Testing of Pulps; Preparation of Laboratory Sheets for Physical Testing; Rapid-Köthen Method. https://www.dinmedia.de/en/standard/din-54358-1/886222.

[39] ISO ISO 5269/2 Pulps—Preparation of Laboratory Sheets for Physical Testing—Part 2: Rapid-Köthen Method. https://www.iso.org/standard/39341.html.

[40] Schreiber A.B., Haimovich J. (1983). [9] Quantitative Fluorometric Assay for Detection and Characterization of Fc Receptors. Methods in Enzymology.

[41] Schäfer J.-L., Meckel T., Poppinga S., Biesalski M. (2023). Chemical Gradients in Polymer-Modified Paper Sheets—Towards Single-Layer Biomimetic Soft Robots. Biomimetics.

[42] Abd El-Mohdy H.L. (2007). Water Sorption Behavior of CMC/PAM Hydrogels Prepared by γ-Irradiation and Release of Potassium Nitrate as Agrochemical. React. Funct. Polym..

[43] Haslach H.W. (2000). The Moisture and Rate-Dependent Mechanical Properties of Paper: A Review. Mech. Time-Depend. Mater..

[44] Hansen C.M., Björkman A. (1998). The Ultrastructure of Wood from a Solubility Parameter Point of View. Holzforschung.

[45] Almgren K.M., Gamstedt E.K., Varna J. (2009). Contribution of Wood Fiber Hygroexpansion to Moisture Induced Thickness Swelling of Composite Plates. Polym. Compos..

[46] Wu N., Hubbe M., Rojas O., Park S. (2009). Permeation of Polyelectrolytes and Other Solutes into the Pore Spaces of Water-Swollen Cellulose: A Review. BioResources.

[47] Wågberg L. (2000). Polyelectrolyte Adsorption onto Cellulose Fibres*—*A Review. Nord. Pulp Pap. Res. J..

[48] Lindström T., Wågberg L., Larsson T., I’Anson S.J. (2005). Review: On the Nature of Joint Strength in Paper*—*A Review of Dry and Wet Strength Resins Used in Paper Manufacturing. Advances in Paper Science and Technology Trans. XIIIth Fund. Res. Symp. Cambridge, 2005.

