# Supplementary material for: Pinecone-Inspired Humidity-Responsive Paper Actuators with Bilayer Structure
Source: Polymers (Basel). 2024 May 15;16(10):1402. doi: 10.3390/polym16101402 (PMC11125993; doi:10.3390/polym16101402)
Supplement: Supplementary file 1 [file polymers-16-01402-s001.zip › polymers-2911572-supplementary.pdf]

polymers

Supporting Information for

Pinecone-Inspired Humidity-Responsive Paper Actuators with Bilayer Structure

David Seelinger [1], Hussam Georges [2], Jan-Lukas Schäfer [1], Jasmin Huong [1], Rena Tajima [1], Christan Mittelstedt [2], Markus Biesalski [1,*]

[1] Ernst-Berl-Institut für Technische und Makromolekulare Chemie, Technische Universität Darmstadt, Peter-Grünberg-Str. 8, 64287 Darmstadt, Germany

[2] Fachgebiet für Leichtbau und Strukturmechanik, Technische Universität Darmstadt, Otto-Berndt-Str. 2, 64287 Darmstadt, Germany

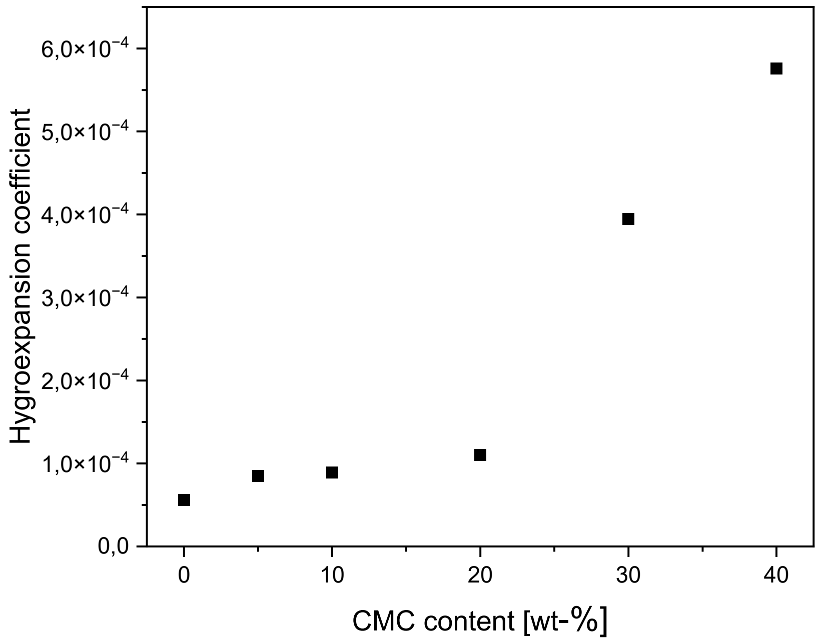

Figure S1. Hygroexpansion coefficients of eucalyptus paper impregnated with varying amounts of CMC.

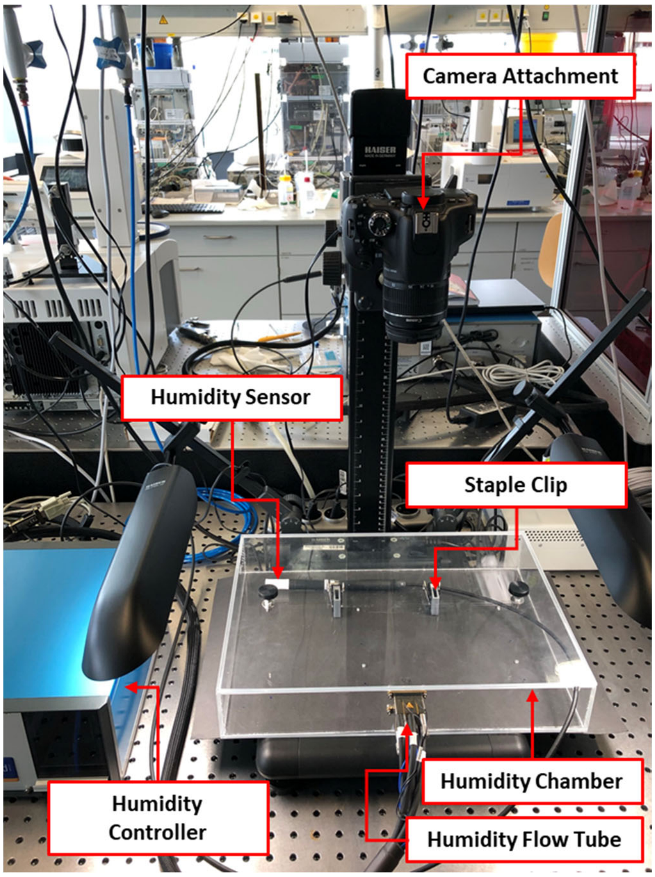

Figure S2. Setup of the PMMA humidity chamber. Paper bilayers are attached to staple clips and arranged horizontally to the bottom of the humidity chamber.

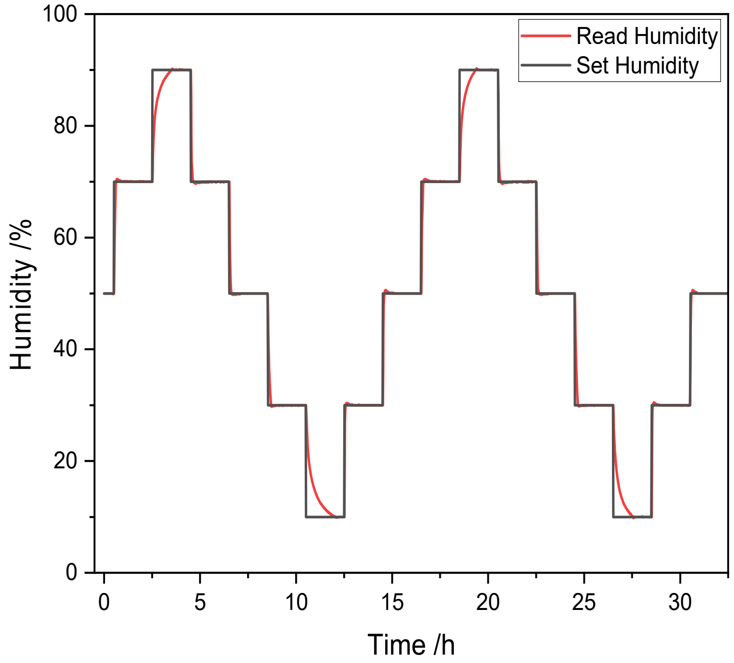

Figure S3. Set and read humidity levels during the humidity treatment for investigation of the bending behavior of paper bilayers.

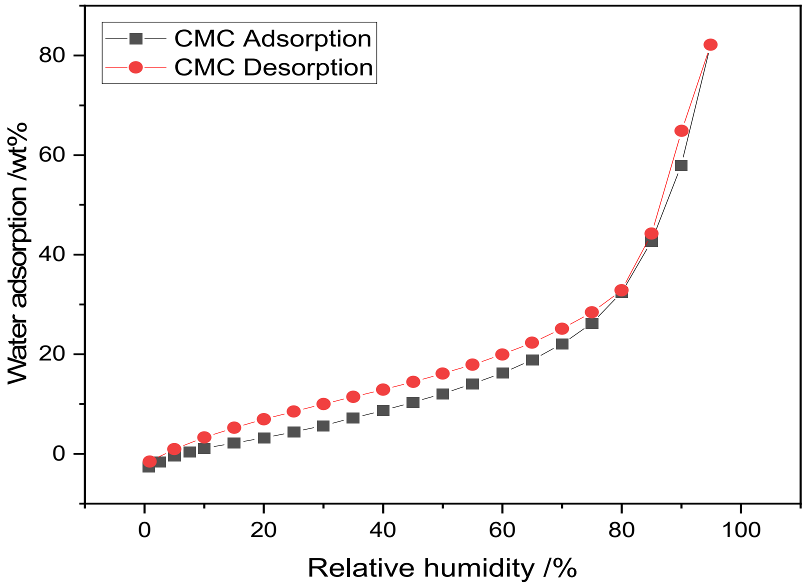

Figure S4. Determination of the dynamic water vapor adsorption and desorption of CMC.

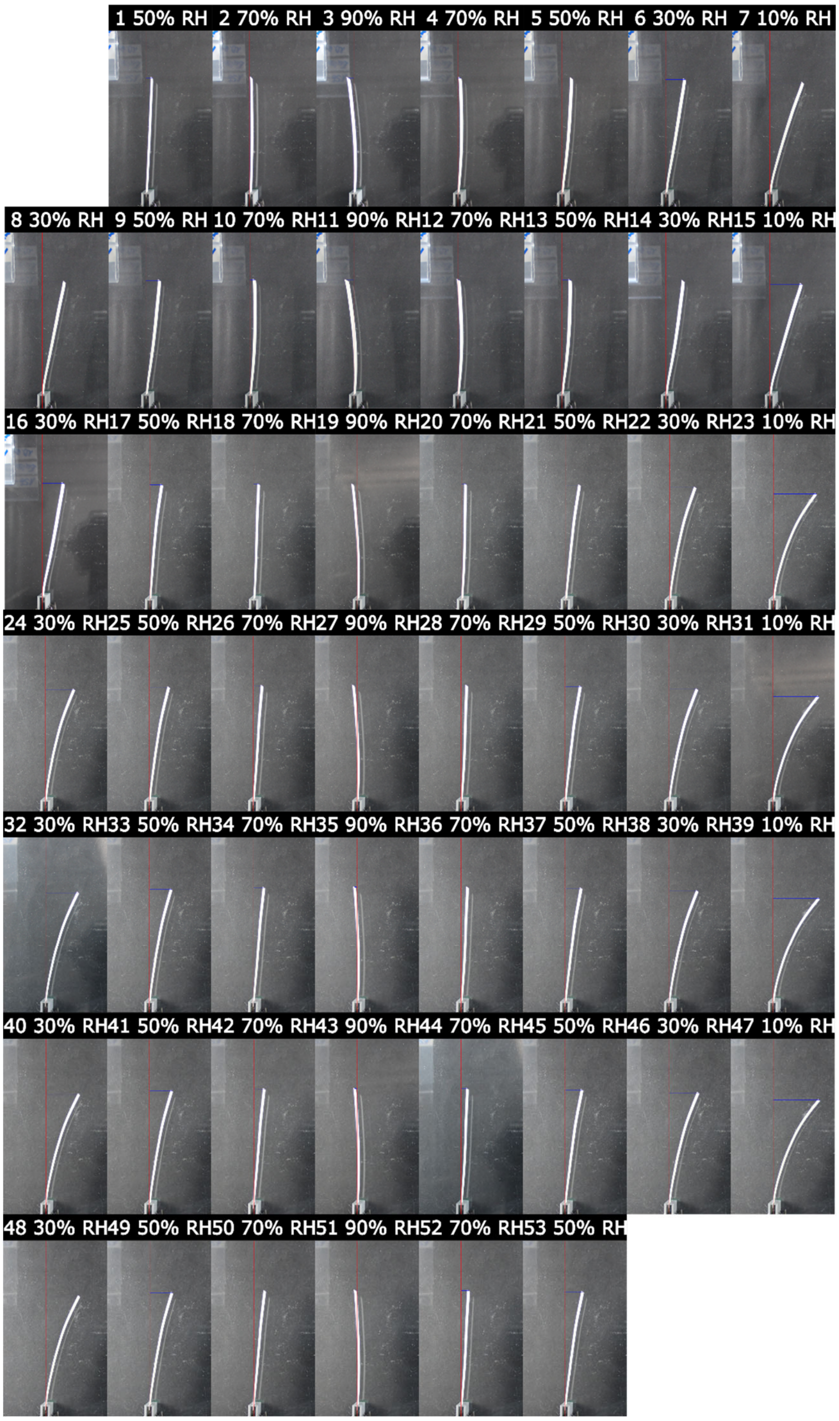

Figure S5. Bending behavior of paper bilayer made from untreated eucalyptus paper stripe laminated to a paper stripe with a content of 10 wt% CMC after several humidity cycles.

Table S1. Summary of applied concentration of aqueous CMC solution to paper samples for the formation paper CMC composites

**CMC content in paper [wt%]**	**Concentration of aqueous CMC solution [wt%]**
1	0,122
2	0,244
5	0,61
10	1,21
20	2,43
30	3,64
40	4,85

Table S2. Young moduli of paper bilayers with varying CMC content

**CMC content**	**Young's modulus [MPa]**
0	1801,55
5 wt% CMC	2199,83
10 wt% CMC	2343,38
20 wt% CMC	2438,98
30 wt% CMC	2744,97
40 wt% CMC	3073,89
